# Single-chain Fv phage display propensity exhibits strong positive correlation with overall expression levels

**DOI:** 10.1186/1472-6750-8-97

**Published:** 2008-12-29

**Authors:** Nathan Scott, Catherine B Reynolds, Michael J Wright, Omar Qazi, Neil Fairweather, Mahendra P Deonarain

**Affiliations:** 1 UCB-Celltech, 208 Bath Road, Slough, Berkshire, SL1 3WE, UK; 2 Department of Paediatrics, University of Texas Medical Branch (UTMB), Galveston, TX 77555, USA; 3 Department of Life Sciences, Faculty of Natural Sciences, Imperial College London, Exhibition Road, London, SW7 2AZ, UK

## Abstract

**Background:**

Single chain Fvs (scFvs) are widely applied in research, diagnostics and therapeutic settings. Display and selection from combinatorial libraries is the main route to their discovery and many factors influence the success of this process. They exhibit low thermodynamic stability, resulting in low levels of premature cytosolic folding or aggregation which facilitates *sec *YEG-mediated translocation and phage in *E. coli*. However, there is little data analysing how this is related to and influenced by scFv protein expression.

**Results:**

We characterised the relationship between overall scFv expression and display propensity for a panel of 15 anti-tetanus toxin scFvs and found a strong positive correlation (Rho = 0.88, p < 0.005) between the two parameters. Display propensity, overall expression and soluble localisation to the periplasm and extracellular fractions were clone specific characteristics which varied despite high levels of sequence homology. There was no correlation between display of scFv or its expression in non-fused (free) form with soluble scFv localisation to the periplasm or culture supernatant. This suggests that divergence in the fate of scFv-pIII and non-fused scFv after translocation to the periplasm accounts for the observed disparity. Differential degrees of periplasmic aggregation of non-fused scFv between clones may affect the partitioning of scFv in the periplasm and culture supernatant abrogating any correlation. We suggest that these factors do not apply to the scFv-pIII fusion since it remains anchored to the bacterial inner membrane as part of the innate phage packaging and budding process.

**Conclusion:**

We conclude that in the absence of premature cytosolic aggregation or folding, the propensity of a scFv to be displayed on phage is directly related to its overall expression level and is thus indirectly influenced by factors such as codon bias, mRNA abundance or putative DNA motifs affecting expression. This suggests that scFvs capable of high overall expression and display levels may not produce high yields of non phage-fused soluble protein in either the periplasmic or extracellular fractions of *E. coli*. This should be considered when screening clones selected from combinatorial libraries for further study.

The nucleotide and amino acid sequences of the anti-tetanus toxin scFvs have been deposited in the EMBL data base: accession numbers-C1: AM749134, C2: AM749135, C3: AM749136, C4: AM749137, C5: AM749138, N1: AM749139, N2: AM749140, N3: AM749141, N4: AM749142, N5: AM749143 J1; AM749144, J2: AM749145, J3: AM749146, J4: AM749147, J5: AM749148.

## Background

During the last two decades single chain Fv (scFv) antibodies have become widely applied in research, diagnostics and therapeutic settings [1]. These recombinant, antigen-binding molecules can be engineered [2,3] to modulate their specificity [4] affinity [5] and pharmacokinetics [6] as well as appending novel effector functions [7,8] Established technology allows investigators to produce large and diverse combinatorial scFv libraries commonly using minor coat protein (pIII) filamentous phage display in *Escherichia coli *(*E. coli*) [9-12]. During production of phage-scFvs, the scFv-pIII fusion is translocated to the periplasmic space and remains anchored in the cytosolic membrane by the C-terminal hydrophobic extension of pIII [13]. The fusion protein then assembles with nascent phage particles as they extrude from the inner membrane.

Overall levels of scFv expression, as with all proteins, are dependant on transcriptional, post-transcriptional and translational level gene regulation. It has been shown that 47% of the variation in *E. coli *protein abundance is accounted for by mRNA abundance alone and codon-bias and codon adaptation indices account for a major proportion of the remaining variation [14,15]. Expression yield often refers to the level of soluble protein produced in the *E. coli *which may be located in the periplasm or in culture supernatant. High thermodynamic stability, high molecular weight, increased hydrophobicity and areas of low sequence complexity are linked to poor soluble protein expression yields [16]. Such properties can lead to proteins being more susceptible to proteolytic degradation [17] aggregation and inclusion body formation in either the cytosol [18] or periplasmic space [19,20]. Disulphide-rich proteins may also be prone to mis-folding once in the periplasm [21].

There is a multitude of research demonstrating that improvements in soluble expression yields in either the periplasm or supernatant can typically be gained by approaches including removal of detrimental hydrophobic residues [22,23], alteration of leader sequence [24], co-expression or over-expression of cytosolic or periplasmic chaperones [25,26] or modifying induction conditions such as inducer concentration, temperature or time [27,28].

Some processes affecting soluble protein expression yield also have bearing upon phage display. In phagemid vector systems [29] the protein-pIII fusion is targeted to the periplasm in the same manner as non-fused protein as they share the same leader sequence. Proteins refractory to *Sec*YEG-mediated periplasmic translocation therefore tend to display poorly in standard phage display systems, elegantly shown for DARPins which have very high thermodynamic stability and are prone to premature folding and aggregation in the cytosol post-translationally [30]. Display levels and periplasmic localisation were drastically improved when the leader sequence was altered for utilisation of the co-translational translocation signal recognition particle pathway which does not allow premature folding or aggregation. This study concluded that when comparing a diverse range of proteins, overall expression level may not correlate with phage display propensity but soluble periplasmic levels do.

There is little data available indicating how overall protein expression level relates to display propensity in the absence of dysfunctional cytosolic-periplasmic protein translocation. The scFv, which is known for its lower thermodynamic stability [31] tends not to prematurely fold in the cytosol of bacteria and is amenable to display and localisation to the periplasm regardless of whether a post or co-translational translocation approach is used [30,32]. Comparing a panel of scFvs in terms of overall protein expression and phage display propensity allows the link between the two characteristics to be investigated.

We used phage display to select scFvs against the recombinant C-terminal domain of the tetanus toxin heavy chain (H_C_) (Scott, N. et al, manuscript in preparation) from a Cambridge Antibody Technology library containing 1.4 × 10^10 ^clones [29]. After two rounds of display we isolated many H_C_-specific clones exhibiting complimentarity determining region (CDR) diversity (Scott *et al*, manuscript in preparation). The scFvs consist of an N-terminal *Sec*YEG translocon signal sequence (VKKLLFAIPLVVPFYAAQPAMA) [29] and C-terminal hexa-histidine (his_6_) and c-myc tags. We sought to compare the phage display properties of 15 anti-Hc scFvs (termed C1–C5, N1–N5 and J1–J5 based on differential epitope binding) in the suppressor strain of *E. coli *XL1-Blue with their facility of expression as non-fused scFvs in the non-suppressor strain HB2151. We observed a strong level of positive correlation between relative overall non-fused scFv expression and relative display level propensity.

## Results and discussion

### Phage display propensity of scFvs is a variable and clone-specific characteristic

**Figure 1:**
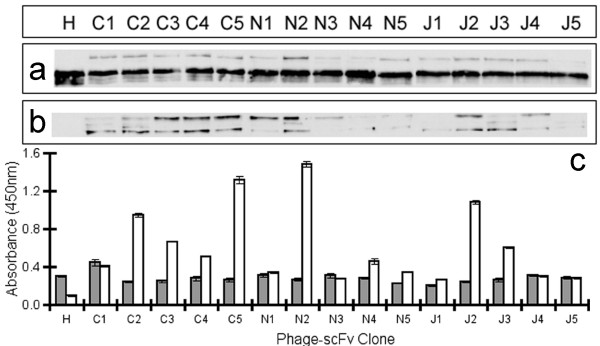
**Analysis of scFv phage display propensity**. Each of the 15 scFv-phage clones and helper phage were produced in *E. coli *(XL1-Blue), equalised, electrophoresed by SDS-PAGE, blotted and probed with (a) anti-pIII antibody to demonstrate equalised phage titre or (b) anti-his_6 _antibody to detect varying levels in display of scFv on phage, as described above. The absolute chemiluminescent signals were measured by densitometry. (c) Binding to undiluted phage coated onto an immunosorbent plate was measured by ELISA to determine levels of pIII and his_6 _using the same antibodies above. ELISA error bars represent standard error from triplicate measurements. Grey bars represent mean anti-pIII signal and white are mean anti-his signal. A representative data set is shown from one batch of each phage-scFv for both the western blot and ELISA methods. Three independently produced batches of phage were analysed.

Phage from *E. coli *XL1-Blue cultures harbouring 15 different anti-Hc scFvs were made, concentrated, purified and quantified by UV spectroscopy (data not shown). Equal numbers of phage particles were blotted onto nitrocellulose following electrophoretic separation. All phage-scFv fusions were probed with either a monoclonal anti-pIII IgG antibody (Figure 1a) or monoclonal anti-his_6 _IgG antibody (Figure 1b). The helper phage only control showed a dominant band migrating at approximately 70 kDa with a variety of commonly observed smaller MW weight products, the result of proteolytic degradation. The 15 anti-Hc phage fusions showed a dominant pIII signal at 70 kDa, that was consistent from clone to clone indicating uniform loading of phage particles displaying each of the anti-Hc scFvs and a less dominant higher MW band migrating at 100 kDa, the density of which varied from clone to clone. Anti-his_6 _immuno-detection for the 15 anti-Hc scFvs varied in intensity from clone to clone and was often observed in two bands at both 70 kDa and 100 kDa. Based on the distribution and signal of pIII and his_6 _bands it is possible to conclude that the 100 kDa bands in Figure 1a solely represent full-length scFv fused to full length pIII. The 70 kDa bands are likely to be due to both wild-type pIII (major species) and pIII appended to the his_6 _tag of the scFv only (minor and indistinguishable). The remainder of the scFv molecule may have been cleaved away by proteolytic activity. It may also be possible that the 70 kDa species represents full length scFv fused to degraded pIII (also indistinguishable). It is also worth noting that previous investigations reveal unexpected electrophoretic migration of pIII due to its high glycine content, extended shape and multi-domain structure leading to differences between calculated and expected molecular weights and this may have contributed to the results observed here [33,34].

Quantitative laser scanning densitometry was used to measure the intensity of the chemiluminescent signal produced by the western blot analyses (data not shown). The total signal obtained from both the 100 kDa and 70 kDa anti-his_6 _signals was used as a measure of display. Regardless of whether the full length scFv is present, detection of the his_6 _tag logically dictates display of that scFv protein on phage. This takes into account degradation (unlike the pIII blot signal). Data analysis was conducted using the AIDA Image Analyser Software and the absolute intensities were corrected for local background signal and normalised for phage loading (using anti-pIII signal) and then used to produce relative rankings of scFv display propensity for three independently produced batches of phage (See additional file 1). An ELISA platform was used to support the display level data obtained by western blot (Figure 1c). Coating of neat, PEG-purified phage (to remove any contaminating soluble scFv) without prior OD normalisation was amenable with this assay platform since variation in titre, as indicated by differences in pIII signal, could be accounted for by obtaining a ratio of his_6 _to pIII OD_450 _values and this was used to produce display rankings.

Three independently-produced batches of the 15 anti-Hc phage-scFvs were analysed by both the western blot and ELISA method described above. Average rankings from 1 (highest displaying scFv) to 15 (lowest displaying scFv) (Table 1) were used to conduct a Spearmans correlation analysis comparing rankings obtained between phage-scFv batches and between the two measurement techniques. Ranks correlated well between batches and techniques (See additional file 1) with clones showing strong anti-his_6 _western blot signals also showing high anti-his_6_:anti-pIII ELISA ratios (e.g clones C3, C4, C5, N2 & J2). Thus display level appears to be a non-batch dependent but clone-specific characteristic and varies markedly between clones in a panel of 15 anti-Hc scFvs.

**Table 1:** Average relative rankings of scFv expression levels in various E. coli compartments and levels of display on filamentous phage.

	Average scFv Rankings				Major Germline Families	
Clone	Supernatant	Periplasm	Spheroplast	Display	VH	VL

C1	6.0 ± 1	9.8 ± 0.8	7.0 ± 1.2	11.7 ± 1.1	VH4	VL3
C2	6.0 ± 0	14.3 ± 0.7	3.0 ± 0.6	6.2 ± 0.5	VH1	VL3
C3	7.5 ± 0.5	3.5 ± 0.5	8.7 ± 2.9	6.3 ± 1.0	VH4	VK1
C4	14.5 ± 0	11.8 ± 1.2	4.0 ± 1.5	4.2 ± 1.2	VH4	VK1
C5	14.5 ± 0	6.0 ± 1	3.7 ± 1.5	2.7 ± 0.3	VH4	VK1
N1	10.0 ± 2	13.8 ± 3.5	10.0 ± 0.6	7.5 ± 2.2	VH2	VK1
N2	4.0 ± 0	4.5 ± 2.2	3.3 ± 1.9	2.8 ± 1.1	VH1	VK1
N3	13.0 ± 0	10.8 ± 0.5	12.0 ± 0	11.5 ± 0.9	VH3	VK1
N4	9.0 ± 0	3.5 ± 0.5	9.3 ± 2.0	12.2 ± 1.1	VH3	VL2
N5	8.3 ± 3.3	4.3 ± 2.7	11.0 ± 1.0	10.7 ± 1.6	VH3	VL1
J1	10.5 ± 0.5	9.0 ± 3	14.7 ± 0.3	12.2 ± 1.1	VH3	VL2
J2	2.0 ± 0	11.8 ± 1.8	4.0 ± 1.5	3.8 ± 0.5	VH1	VK1
J3	2.0 ± 1	3.8 ± 2.2	5.3 ± 1.3	6.5 ± 1.3	VH3	VL2
J4	2.0 ± 1	3.5 ± 1.5	10.0 ± 1.0	11.3 ± 0.7	VH6	VL2
J5	10.8 ± 0.7	10.0 ± 1	14.0 ± 0.6	11.3 ± 1.5	VH3	VL2

**Figure 2:**
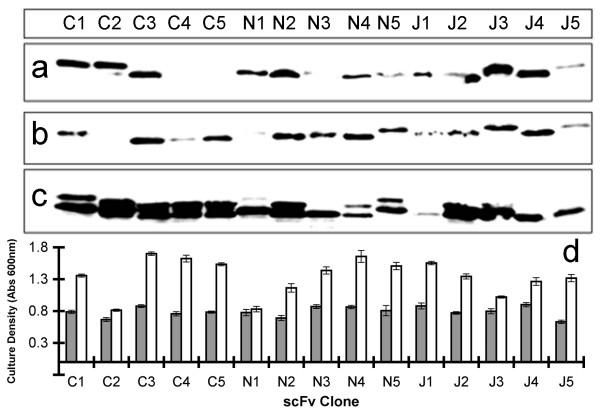
**Compartmental analysis of overall relative levels of scFv expression in Escherichia coli**. Each of the 15 scFv clones were produced in *E. coli *(HB2151). The cultures were equalised for bacterial density and the supernatant, periplasmic and spheroplast fractions were electrophoresed by SDS-PAGE and blotted. Anti-his_6 _antibody was used to probe for the scFvs, developed by chemiluminescence. (a) supernatant fraction, (b) periplasmic fraction and (c) spheroplast *E. coli *pellet. The blot is representative of three independent experiments conducted on independently produced samples. (d) Pre-induction (grey bars) and final culture densities (white bars) measured by OD_600 _are also shown and represent mean values from three independent experiments.

Densitometry data derived from the western blots shown in Figures 1 and 2 were used to rank scFvs from 1 to 15 in order of highest to lowest levels of scFv respectively. Rankings from repeat experiments were averaged and are shown here along with standard error for the overall expression and display levels. The average relative spheroplast scFv level rankings were used as an indicator of overall expression level and to designate the scFvs into three groups of either "high (average ranking ≤ 5; **bold **numbers)", "medium (average ranking ≥ 5 but ≤ 10; *italicised *numbers) " or "low" (average ranking ≥ 11; No Shading) expression. Rankings for phage display propensity were also used to assign similar groupings. The major V_H _and V_L _germline designations for each scFv are also shown for each clone.

This variability in display level was not unexpected given that these clones were derived after only 2 rounds of selection on the Hc antigen (Scott et al, manuscript in preparation). Such differences in display level may have implications in selections such that scFvs exhibiting a higher display level may be preferentially enriched in the absence of vastly differing affinities. Given that scFvs have been shown to be relatively resistant to premature cytosolic folding/aggregation [31,32], we hypothesised that the display level variation shown here is due to variation in the overall expression level of the scFvs with the reasonable assumption that facility for translocation to the periplasm is uniform amongst the 15 clones.

### Overall scFv expression does not correlate with soluble levels in the periplasm and culture supernatant

We found that the overall level of scFv expression defined as levels measured by western blot in the spheroplast fraction of *E. coli *HB2151 does not correlate with soluble levels in the periplasmic or culture supernatant fractions after 16 hr culture induction.

Spheroplast, periplasmic and supernatant fractions were isolated from the cultures at the end of the 16 hr induction period following normalisation of the culture density of each clone. Fractionated samples were electrophoresed and transferred onto nitrocellulose for probing with an anti-his_6 _antibody (Figure 2a–c). The supernatant and periplasmic fractions of each anti-Hc scFv exhibited a single band at approximately 30 kDa indicating fully processed scFv. The spheroplast fractions of most scFvs contained doublet bands at approximately 30 kDa, likely to represent both N-terminally processed and unprocessed scFv in addition to smaller breakdown products. The absolute signals vary markedly between scFv clones in each of the three fractions. Quantitative laser scanning densitometry measurements of western blot chemiluminescence were acquired (data not shown) and used to rank scFvs from 1 (high) to 15 (low) in order of densitometry signals for levels of scFv present in the *E. coli *spheroplast, periplasmic lysate and culture supernatant. The overall rankings were reproducible between data sets (See additional file 1) for the same *E. coli *compartment indicating that overall scFv expression (spheroplast level) and localisation to the periplasm and culture supernatant in soluble form are clone-specific characteristics, that vary from clone to clone.

Average rankings were produced for each compartment using independently produced data sets (Table 1) and these were used to conduct a Spearmans correlation test in order to determine if relative levels of scFv present within the three fractions correlate with each other for each clone. For n = 15, a Rho value greater than 0.441 indicates positive correlation with a p-value of less than or equal to 0.05. However, comparison of supernatant scFv levels with periplasmic or spheroplast levels yielded coefficients of 0.38 and 0.37 respectively. Comparison of periplasmic and spheroplast levels yielded a coefficient of 0.13. These data suggest there is no significant correlation (p > 0.05) between the overall expression level of a scFv molecule as defined by its spheroplast level, and its relative soluble levels within the periplasmic space or culture supernatant.

Interestingly in three independent experiments the final culture density, as determined by OD_600 _after a 16 hr induction, reached a value reproducibly characteristic to the clone being studied ranging from 0.8 for C2 to 1.7 for N4 (Figure 2d). Relative increases in culture density following induction were calculated using the mean culture density data shown in Figure 2d. The relative increases were used to rank the scFvs from 1 (highest fold increase) to 15 (lowest fold increase) and used to determine Spearmans correlation coefficients in pair-wise comparisons with spheroplast, periplasmic and culture supernatant scFv level rankings. Although there was no detectable correlation between relative increase in culture density ranking and spheroplast or periplasmic scFv level rankings, there was a moderate but statistically significant negative correlation (Rho = -0.55, p < 0.05) when compared with culture supernatant scFv levels. Thus scFv clones that exhibit a low relative level of culture supernatant localisation allow the bacteria harbouring them to attain a higher culture density than other scFvs.

Given that scFvs are not prone to cytosolic aggregation, the lack of correlation between the spheroplast and periplasmic levels of scFv may be explained by differential toxicity once in the periplasm. Our culture density data suggests that some scFvs are more toxic than others and that clones with a high level of supernatant localisation hinder bacterial growth following induction, perhaps through lytic mechanisms or destabilisation of the outer membrane. This non-uniform differential soluble scFv localisation therefore directly influences any correlation between supernatant levels and periplasm, and supernatant/periplasm with spheroplast levels. Differing levels of toxicity may be due to periplasmic aggregation and hence inclusion body formation, proteolytic breakdown or mis-folding due to inappropriate disulphide bond formation which has been observed previously for scFvs [23,35].

### Phage display propensity exhibits strong positive correlation with overall scFv expression Levels

**Figure 3:**
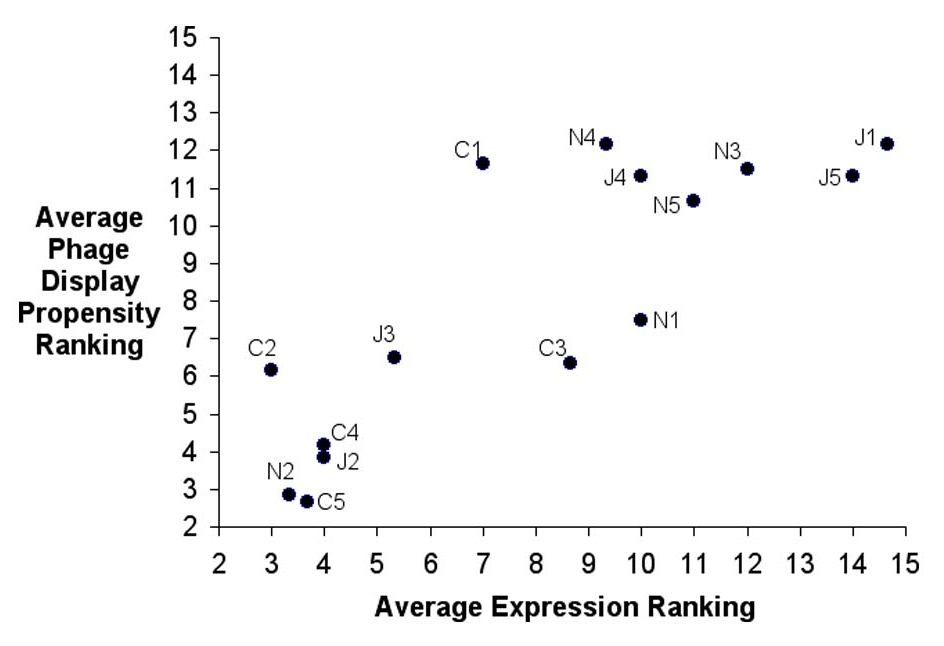
**Overall levels of scFv expression exhibit strong positive correlation with phage display propensity**. Average relative rankings obtained for overall scFv expression in the spheroplast fraction of *E. coli *are displayed on a scatter-plot in relation to average relative phage display propensity rankings for each individual clone. A ranking of 1 represents a high relative level of overall expression or phage display propensity and a ranking of 15 represent a low relative level of overall expression or phage display propensity. The plot shows a strong positive correlation between expression ranking and display level ranking. Each scFv x-axis co-ordinate represents the average ranking from 3 independent experimental observations of overall scFv expression (Table 1). Each scFv y-axis co-ordinate represents the average ranking from 6 independent experimental observations of display level (three ELISA and three western blots) carried out on three batches of phage-scFv (Table 1). The Spearmans coefficient of rank correlation Rho coefficient for this association is 0.88 representing strong positive correlation with a p-value < 0.005. Standard errors are not conventionally used for averages of ranks. However they have been calculated (Table 1) and are not shown graphically for purposes of clarity.

The average relative scFv rankings for display level propensity were compared with the expression analysis of non-fused scFv. We found that display level ranking does not correlate with soluble scFv level rankings in either the periplasm (Rho = 0.25; p > 0.05) or supernatant (Rho = 0.20; p > 0.05). However, we found a strong positive correlation (Rho = 0.88; p < 0.005) between display level ranking and overall scFv expression ranking as defined by relative spheroplastic levels, illustrated in Figure 3. Based on the ranking data, scFv clones were grouped into 'high', 'medium' or 'low' for phage display propensity and overall expression levels (Table 1). Display ranking was achieved using two independent methods on three independent batches of material and was remarkable consistent (See additional file 1). The average ranking was at worst, only varied by 2 places (clone N1). Total expression ranking was also very consistent with clone C3 being the most variable. Taking all factors into account, we believe this to be a robust and strong correlation.

This interesting correlation provides insight into the possible mechanism leading to a lack of correlation between overall scFv expression and soluble levels in the periplasm and supernatant. Assembly of pIII-scFv fusion proteins into phage particles is dependant upon their translocation to the periplasm [30]. Since overall expression level correlates strongly with display level, it may be concluded that few of these scFv clones exhibit a marked level of premature folding or degradation in the bacterial cytosol that may hinder their translocation to the periplasm. Any degree of premature folding may be assumed to be uniform amongst the 15 clones since the only factor influencing display level is simply the absolute level of scFv expressed in the bacterial cytosol. Given that scFv expression in both pIII-fused and non-fused forms share the same secretary signal sequence it can be assumed that both are trafficked to the periplasm in an identical manner. We would therefore expect levels of soluble scFv in the periplasm to correlate with overall expression level and display level. This was not the case.

A hypothetical explanation for the observed disparity therefore potentially lies in the fact that the fates of pIII fused and non-fused scFvs diverge once the proteins are translocated across the inner membrane of *E. coli*. The pIII-scFv fusion remains anchored in the inner membrane via the C-terminal domain of pIII [13] whereas free scFv remains in the periplasmic space where it is subject to potential mis-folding or aggregation. We propose that differential levels of aggregation between clones at this stage leads to varying degrees of membrane destabilisation and therefore leaking of scFv into the supernatant.

### Codon usage indirectly influences scFv phage display propensity

**Figure 4:**
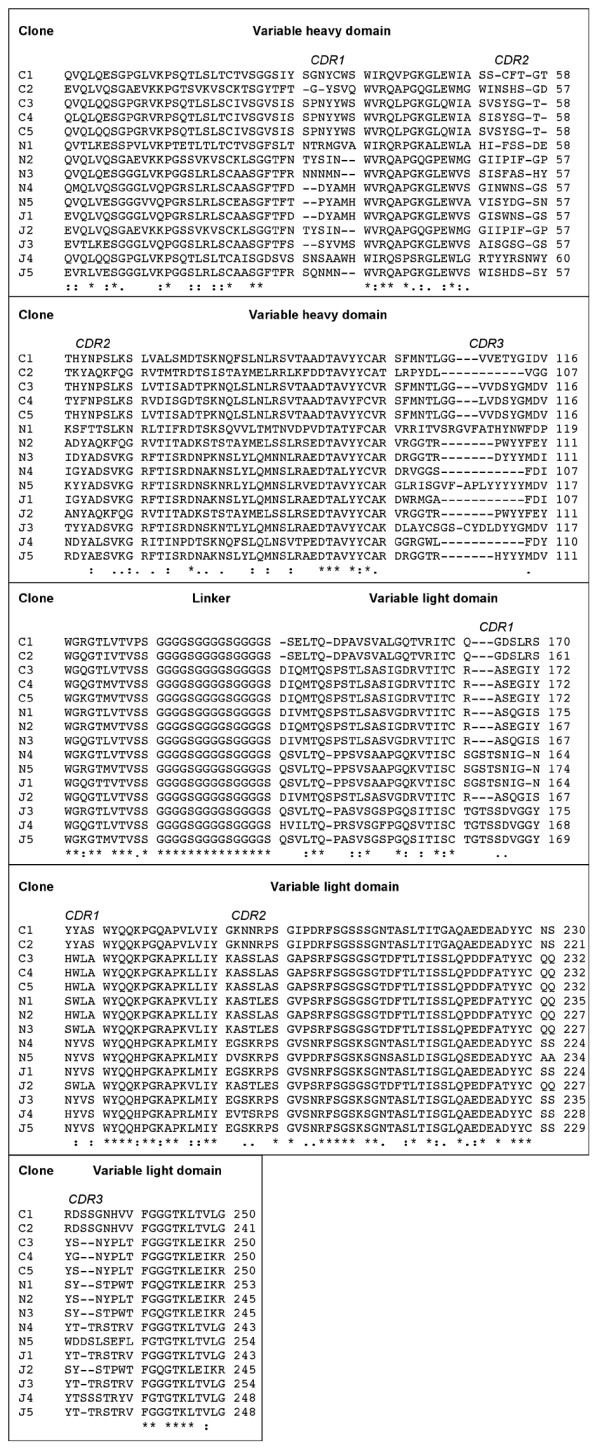
**Annotated amino acid sequence alignment of the 15 anti-Hc scFvs**. The amino acid sequences of 15 anti-Hc scFvs C1–C5, J1–J5 and N1–N5 were aligned using ClustalW [45]. The inter-domain linker (G_4_S)_3 _joins the N-terminal V_H _domain to the C-terminal V_L _domain of the scFv. Within each of the V_H _and V_L _domains, there are three areas of increased diversity indicating the hypervariable complimentarity determining regions (labelled). Identically-conserved residues are labelled (*), conserved residues are labelled (:) and similar residues are labelled (.) The protein and corresponding DNA sequences (See additional file 2) have been deposited in the EMBL database.

Figure 4 shows the amino acid sequences of the 15 anti-tetanus toxin Hc scFvs aligned by homology. The major germline constituents of the scFv V_H _and V_L _domains were determined (Table 1) in order to evaluate whether particular germline sequences are more amenable to expression in *E. coli *and hence display on phage. It seems that scFvs exhibiting a high level of display and expression often harbour a VK1 derived V_L _domain and those exhibiting low expression and display tend to harbour a VH3 derived V_H _domain. These observations support earlier work where Ewert et al [31] analysed total soluble expression of individual and paired V-domains. Here also, VH3 subtype was best and most soluble VH domain and VK1 was also ranked highly. It was not possible to elucidate definite D and J segment germline designations for the scFv V_H _and V_L _domains since they appear to have undergone significant affinity maturation. This is not unexpected as the library used was derived from a pool of individuals many of whom were likely to have been immunised against tetanus, leading to antibody affinity maturation.

The link between total protein expression and phage display is supported by bioinformatics analyses. A number of factors influence translation efficiency, codon usage being an important parameter [14,15]. One codon usage index is the codon adaptation index (CAI) [36] which was determined using the codonW programme [37]. Ranking of the CAI correlates with overall expression and hence phage display (Rho = 0.68 p < 0.01). At the nucleotide level, the scFv sequences are similar (See additional file 2) and the CAI covers a small range, suggesting that minor differences in codons could account for large differences in display and expression. The Improbizer program [38] was used to search for DNA motifs which could be linked to high expression. However, many motifs were identified and given the similar nucleotide backgrounds, it was impossible to deduce which ones could be of significance and which ones were signatures of the V-gene sequences. A similar protein sequence analysis failed to identify any protein motifs which correlate with phage display propensity. This confirms that phage display of scFvs is closely and significantly linked to overall protein expression and not post-translational protein-orientated events such as folding. There is a great deal of research detailing protein sequence motifs which affect functional protein expression, particularly recombinant antibodies [23,39]. This is likely to affect the levels of scFv found in the supernatant. Antibody V-domains are generally homologous with a consensus homology of approximately 50% [40]. The data in this study comprises V-domains ranging from highly similar sequences to around 60% homology (Fig. 4). Given that even a single residue change can influence major biophysical properties [23], making observations with highly similar sequences is still valid for this comparative analysis.

## Conclusion

In conclusion, when premature protein folding in the bacterial cytosol is not a factor, phage display levels correlate with overall expression level. Higher levels of synthesised protein result in greater levels of inner membrane translocation and incorporation into phage particles. Therefore, factors influencing overall expression indirectly influence phage display propensity. This suggests that better scFv display could be achieved in codon-optimised strains of *E. coli *such as those commercially available or in vectors with very strong promoters such as the T7-promoter system. However, high levels of overall expression may not necessarily imply high levels of soluble localisation to the periplasm or supernatant. This may influence selection of clones for further study after phage display suggesting that a parallel screen of periplasmic or supernatant levels should be conducted in order to select clones that exhibit a desirable high soluble yield in addition to phage ELISAs. The work also shows how scFv display level and expression can vary considerably amongst clones despite an overall high level of sequence homology.

## Materials and methods

### Production and isolation of phage-scFv fusions

A description of the selection and full characterisation of these 15 anti-tetanus toxin scFvs is in preparation (Scott, N et al, manuscript in preparation). All scFvs were selected on plastic immobilised recombinant tetanus toxin Hc-domain using Nunc immunotubes at high antigen concentrations. This allowed selection of a diverse array of binders with no selection pressure on affinity, display or expression.

The amber suppressor XL1-Blue strain of *Escherichia coli *(Stratagene) was transformed [41] with the phagemid vector pCANTAB6 [29] encoding each of the fifteen anti-Hc scFvs C1–C5, N1–N5 and J1–J5 under the control of the *lac *promoter. Individual transformant colonies of each scFv clone were selected and grown for 16 hours in a 5 ml culture of 2TY [41] containing 100 μg/ml carbenicillin, 15 μg/ml tetracycline and 1% glucose, with shaking at 250 rpm. The cultures were sub-cultured, after removal of old medium, at a 1:100 ratio into 50 ml of fresh medium and grown at 37°C with shaking at 250 rpm. At an OD_600 _of 0.4–0.5, the phagemid containing bacteria were infected with VCSM13 helper phage (Stratagene) at a multiplicity of infection of 20:1 [42]. Infection was allowed to take place for 30 minutes at 37°C without shaking followed by a further 30 minutes at 37°C with shaking at 200 rpm. The bacteria were isolated by centrifugation and resuspended in 50 ml of 2TY containing 100 μg/ml carbenicillin and 50 μg/ml kanamycin. The cultures were incubated overnight at 30°C with shaking at 300 rpm. The culture supernatants were clarified by centrifugation and filtered through a 0.44 μm membrane. A third volume of ice-cold 20% polyethylene glycol 8000 containing 2.5 M NaCl was mixed with the phage containing supernatant and the solution incubated for 2 hours at 4°C. Precipitated phage were isolated by centrifugation at 4000 rpm for 1 hour at 4°C and resuspended in commercial phosphate buffered saline (PBS) (pH 7.4) to a volume one tenth the original culture volume. A final centrifuge step at 13,000 rpm was used to remove any debris. Phage titre was estimated for each phage-scFv clone using UV spectroscopy (OD_270 _= 1 is equivalent to 1.1 × 10^13 ^phage particles per millilitre) [43].

### Expression of non-fused scFv and isolation of spheroplast, periplasmic and supernatant fractions

The DNA encoding the fifteen anti-Hc scFvs in phagemid pCANTAB6 were transformed into the HB2151 (GE Healthcare, UK) strain of *E. coli *which produces only scFv rather than a mixture of non-fused scFv and scFv-pIII fusion since translation terminates at the amber TAG codon separating the two genes. The clones were grown for 16 hours at 37°C in 2TY broth containing 1% glucose and 100 μg/ml carbenicillin with shaking at 250 rpm. After removal of old medium the cells were sub-cultured at a 1:100 ratio into 10 ml of fresh medium and grown to an OD_600 _of between 0.6 and 0.8 at 37°C with shaking at 250 rpm [42]. At this point the bacteria were isolated by centrifugation and resuspended in 10 ml of 2TY medium containing 100 μg/ml carbenicillin and 1 mM IPTG to induce expression of the non-fused scFv genes. The cultures were incubated overnight at 30°C with shaking at 300 rpm. The OD_600 _was measured at the end of this period and the culture volumes adjusted with 2TY broth in order to normalise the bacterial density to that of the lowest clone. The cultures were centrifuged at 4000 rpm for 1 hour at 4°C and the supernatant retained. The bacterial pellets were re-suspended in ice-cold periplasmic extraction buffer containing 500 mM sucrose 100 mM Tris-HCl, 1 mM EDTA pH 8.0 [44]. The volume of buffer used was one-tenth the final OD normalised volume. The resuspended bacteria were mixed by vortexing for 10 seconds every 5 minutes for 20 minutes in order to break open the outer membrane of the bacteria. Spheroplasts were then isolated by centrifugation at 13,000 rpm for 30 minutes at 4°C and the supernatant (periplasmic fraction) retained. The spheroplast containing pellet was re-suspended in distilled H_2_0 at one-tenth the volume of OD normalised culture volume.

### Measurement of phage display levels and non-fused scFv expression using western blot with quantitative laser scanning densitometry analysis

For measurement of phage display propensity, equal numbers of phage displaying each of the 15 scFv clones and a helper phage control were subject to reducing SDS-PAGE and subsequently electroblotted onto nitrocellulose. Each clone and the helper phage were probed with a murine anti-pIII antibody (Clone 10C3, MoBiTec, Germany-which recognises a 10 residue C-terminal epitope, thus suitable for investigating degraded scFv-pIII fusion proteins) followed by a goat anti-mouse HRPO secondary. Simultaneously the samples were probed with HRPO conjugated murine anti-his_6 _antibody (Sigma). The detection of pIII is a measure of total phage present and the measurement of the his_6 _tag indicates levels of scFv displayed. In the case of non-fused scFv, the supernatant (5 μl), periplasmic (0.5 μl) and supernatant (0.5 μl) fractions were subjected to reducing SDS-PAGE and electroblotted onto nitrocellulose. Probing with anti-his_6 _HRPO was used to measure the scFv levels. In both the case of measuring levels of scFv fused to phage and of non-fused scFv, western blots were developed using the ECL plus western blotting detection system. Chemiluminescent signals were detected and measured using quantitative laser scanning densitometry [LAS3000 CCD Imaging System] (Fujifilm, Japan) facilitated with the AIDA Biopackage image analyser software (Raytest, Germany). Absolute signal intensities derived from the chemiluminescent signal were corrected for local background signal and used to produce a relative ranking of scFv display level and levels of non-fused scFv in spheroplast, periplasm and supernatant.

### Measurement of scFv display level by ELISA

As an additional measurement of scFv levels on phage, an ELISA methodology was used. Undiluted phage (100 μl) were coated onto 96-well Maxisorp Nunc-Immunoplates. Following a blocking step with Marvel™ dried milk powder pIII and his_6 _immunodetection were facilitated with the antibodies used during the western blot procedure. The assay was developed using BM-Blue POD substrate and stopped with 1 M HCL. Binding signal (OD_450_) was measured using a SpectraMax 340PC plate reader. A ratio of pIII to his_6 _OD_450 _values was used as an indication of relative display level to account for variation in phage titre and rankings were produced accordingly.

### Statistical analyses

Statistical assessment including derivation of means and standard errors was carried out using the Sigma Plot Software Version 8.0 (Systat Software Inc., London). Comparison of scFv rankings between different sample sets was facilitated through calculation of the Spearmans Coefficient of Rank Correlation parameter (Rho) which shows the degree of correlation and its statistical significance.

## Abbreviations

OD_600_: Optical density at 600 nm; HRPO: Horse-radishperoxidise; ELISA: Enzyme linked immuno-sorbant assay; scFv: Single chain Fv; *E. coli*: *Escherichia coli*; H_c_: Tetanus toxin heavy chain C-terminal domain; his_6_: Hexahistidine epitope tag; pIII: Minor protein 3 of phage.

## Authors' contributions

OQ and MW originally selected the anti-toxin scFvs under the direction of NF and MD. NS carried out the scFv-phage identifications, preparations, scFv expression, display analyses, statistical analyses under the direction of MD. NS and MD drafted the manuscript. CR carried out some of the scFv-phage preparations and display analyses. All authors read, contributed to and approved the final manuscript.

## Supplementary Material

Additional file 1 **Individual rankings from the independent batches of scFv-phage in the different experiments.** This shows the consistency and reliability of the data used to derive the correlations. Click here for file

Additional file 2 **Annotated nucleotide sequence alignment of the 15 anti-Hc scFvs.** The DNA sequences of fifteen anti-Hc scFvs C1–C5, J1–J5 and N1–N5 were aligned using ClustalW [45]. Conserved bases are labelled (*). Click here for file
