# Supplementary material for: Single-chain Fv phage display propensity exhibits strong positive correlation with overall expression levels
Source: BMC Biotechnol. 2008 Dec 29;8:97. doi: 10.1186/1472-6750-8-97 (PMC2630973; doi:10.1186/1472-6750-8-97)
Supplement: Additional file 1 — Individual rankings from the independent batches of scFv-phage in the different experiments. This shows the consistency and reliability of the data used to derive the correlations. [file 1472-6750-8-97-S1.doc]

**Table 2**

***Individual rankings from the independent batches of scFv-phage in the different experiments***.

ELISA Ranking Anti-His-6 blot Ranking Supernatant Expr. Periplasmic Expr. Spheroblast Expr

| Repeats | 1 | 2 | 3 | 1 | 2 | 3 | 1 | 2 | 1 | 2 | 1 | 2 | 3 |
| --- | --- | --- | --- | --- | --- | --- | --- | --- | --- | --- | --- | --- | --- |
| C1 | 14 | 13 | 10 | 9 | 15 | 9 | 7 | 5 | 10.5 | 9 | 7 | 5 | 7 |
| C2 | 4 | 6 | 7 | 6 | 7 | 7 | 6 | 6 | 13.5 | 15 | 5 | 3 | 4 |
| C3 | 5 | 10 | 9 | 4 | 5 | 5 | 8 | 7 | 3 | 4 | 13 | 14 | 4 |
| C4 | 7 | 4 | 8 | 1 | 1 | 4 | 14.5 | 14.5 | 10.5 | 13 | 9 | 7 | 2 |
| C5 | 2 | 2 | 4 | 3 | 3 | 2 | 14.5 | 14.5 | 7 | 5 | 10 | 6 | 4 |
| N1 | 11 | 8 | 15 | 8 | 2 | 1 | 12 | 8 | 13.5 | 14 | 11 | 11 | 9 |
| N2 | 1 | 1 | 1 | 2 | 4 | 8 | 4 | 4 | 8 | 1 | 8 | 2 | 1 |
| N3 | 15 | 11 | 11 | 12 | 8 | 12 | 13 | 13 | 13.5 | 8 | 14 | 12 | 12 |
| N4 | 8 | 15 | 14 | 11 | 11 | 14 | 9 | 9 | 4 | 3 | 2 | 9 | 13 |
| N5 | 9 | 7 | 6 | 15 | 14 | 13 | 5 | 11.5 | 1.5 | 7 | 12 | 10 | 10 |
| J1 | 10 | 14 | 13 | 13 | 13 | 10 | 11 | 10 | 6 | 12 | 15 | 15 | 15 |
| J2 | 3 | 3 | 3 | 5 | 6 | 3 | 2 | 2 | 13.5 | 10 | 3 | 1 | 6 |
| J3 | 6 | 6 | 2 | 7 | 12 | 6 | 1 | 3 | 1.5 | 6 | 1 | 4 | 8 |
| J4 | 13 | 13 | 12 | 10 | 9 | 11 | 3 | 1 | 5 | 2 | 6 | 8 | 11 |
| J5 | 12 | 12 | 5 | 14 | 10 | 15 | 10 | 11.5 | 9 | 11 | 4 | 13 | 14 |

This shows the consistency and reliability of the data used to derive the correlations.
