# Supplementary figures and images for: Single-chain Fv phage display propensity exhibits strong positive correlation with overall expression levels
Source: BMC Biotechnol. 2008 Dec 29;8:97. doi: 10.1186/1472-6750-8-97 (PMC2630973; doi:10.1186/1472-6750-8-97)

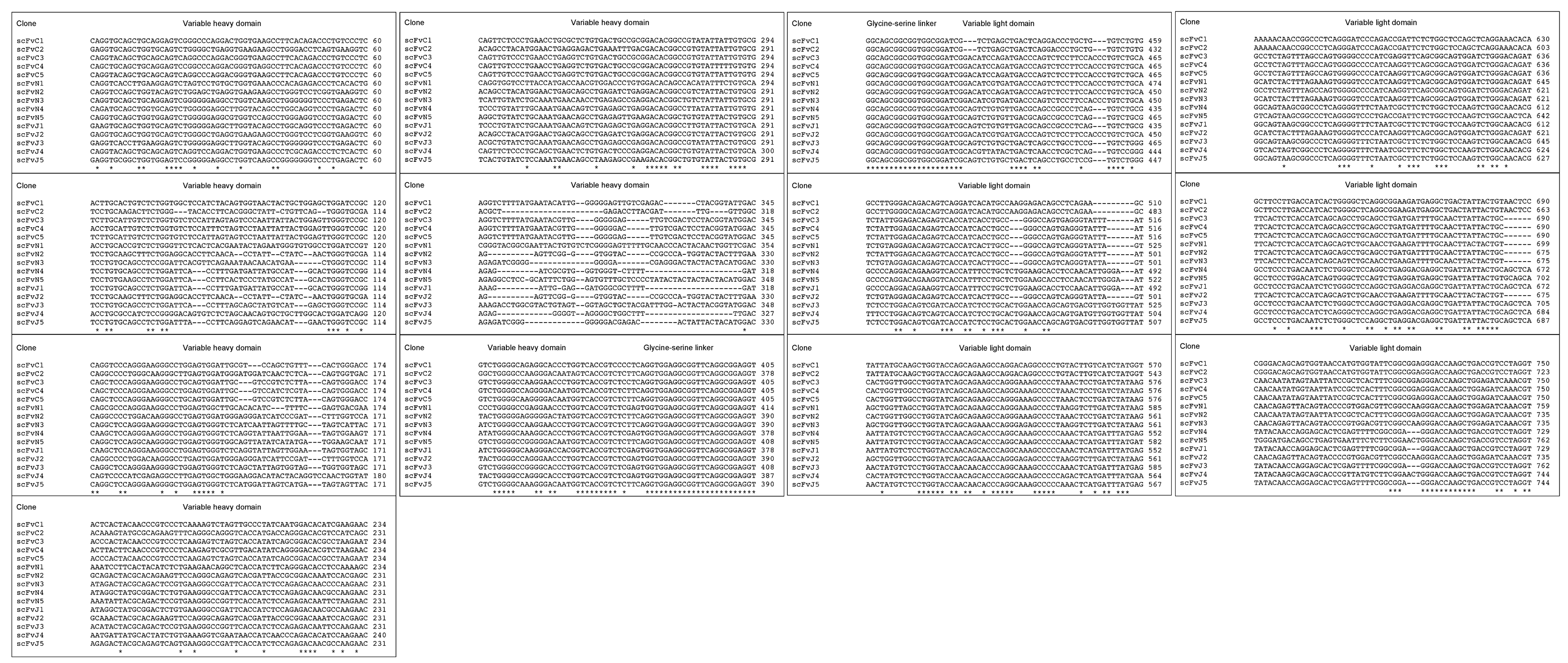

Supplement: Additional file 2 — Annotated nucleotide sequence alignment of the 15 anti-Hc scFvs. The DNA sequences of fifteen anti-Hc scFvs C1–C5, J1–J5 and N1–N5 were aligned using ClustalW [45]. Conserved bases are labelled (*). [file 1472-6750-8-97-S2.tiff]
